# Supplementary material for: Androgen receptor signaling regulates the transcriptome of prostate cancer cells by modulating global alternative splicing
Source: Oncogene. 2020 Aug 20;39(39):6172–89. doi: 10.1038/s41388-020-01429-2 (PMC7515832; doi:10.1038/s41388-020-01429-2)
Supplement: Supplementary file 1 — Supplementary Material [file 41388_2020_1429_MOESM1_ESM.pdf]

Supplementary Figure 1

Androgen Receptor Signaling Regulates the Transcriptome of Prostate Cancer Cells by Modulating Global Alternative Splicing.

Discovery
Human Transcriptome Array
LNCaP (Casodex vs DHT)
Differentially Expressed Genes
Alternative splicing events

Filtering criteria for RT-PCR validation in LNCaP, 22RV1, and PC3 Cells

1) Genes that are not differentially regulated
2) Splicing Events with FDR cut off of 0.05 and SI of >= |2|.
3) Events with evidence from probes mapping to alternatively spliced region and junction surrounding it.
4) Genes with a known biological role in cancer.

Functional Analysis

1) Identify splicing within a curated list of 100 prostate cancer relevant genes.
2) Validation of functional impact of alternative splicing using TCGA data.
3) GO pathway analysis to study the biological relevance of genes alternatively spliced in response to treatment with AR inhibitor.

Independent Validation
RNASeq (rMATS analysis)
GSE110903, GSE110903 & in-house data
LNCaP: Enzalutamide vs DMSO
DHT vs DMSO
MDA-PCA-2B : AR KD vs control

Concordance between affy and RNAseq analysis
Identify alternative splicing analysis and events that overlap with human transcriptome array

Functional Analysis

1) Maser analysis to study the functional domains which are alternatively spliced in response to AR inhibitors.
2) rMAPs analysis to identify androgen regulated RNA Binding Proteins binding in and around alternatively spliced exons.

Identifying potential splicing events associated with prostate cancer disease
Dataset: GSE80609
8 benign prostate hyperplasia (BPH),
16 localized prostate cancer (L.PC),
9 advanced prostate cancer (advPC),
12 castrate-resistant prostate cancer (CRPC),
four pairs of advPC and CRPC samples from the same patient

Androgen regulated alternatively spliced genes associated with progression of prostate cancer
Identify splicing event significant across all different stages of prostate cancer and those induced by pharmacological and genomic inhibition of AR in prostate cancer cells

Functional Analysis
Validation of functional impact of alternative splicing using TCGA data

Supplementary Figure 2

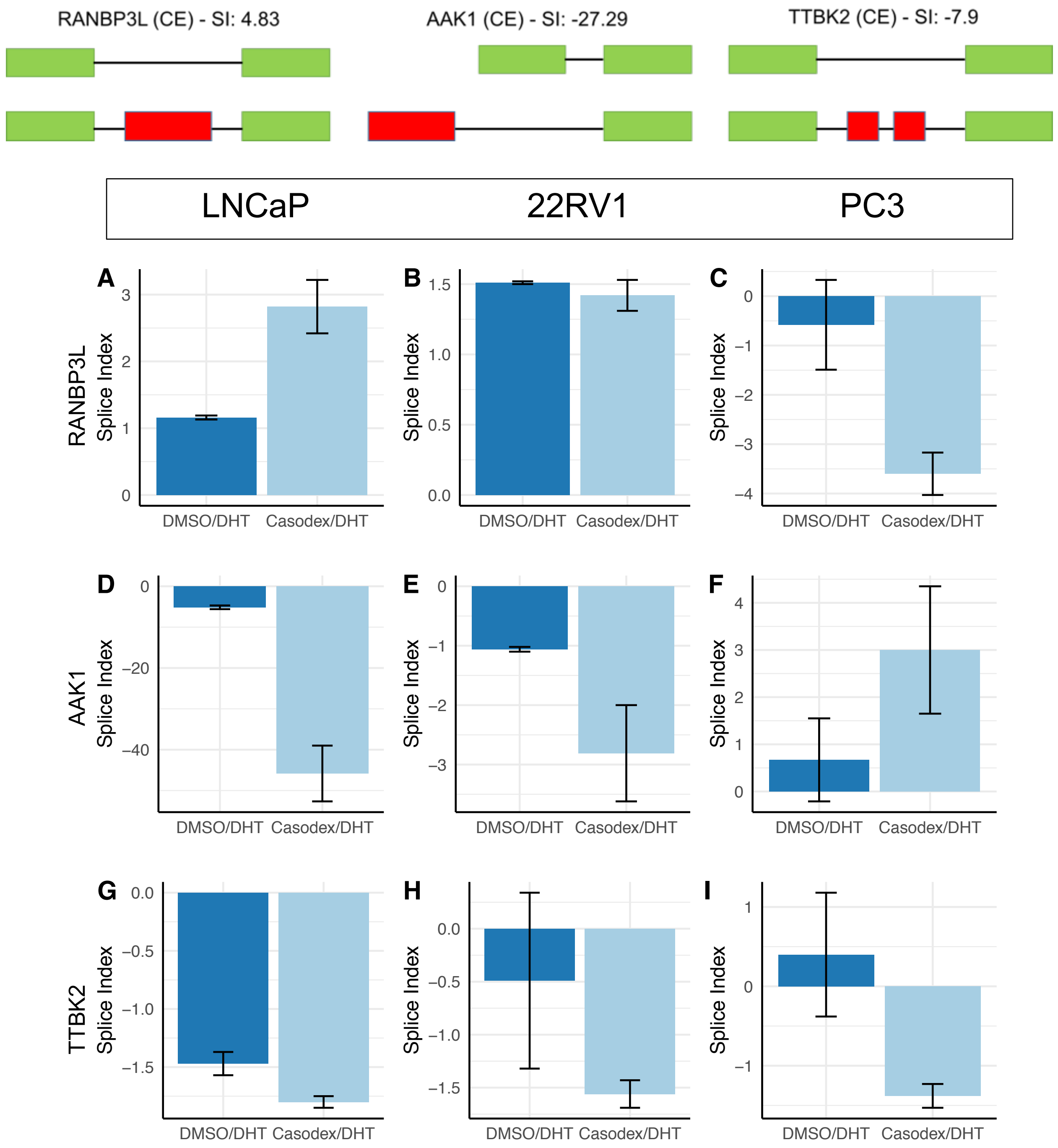

Supplementary Figure 3

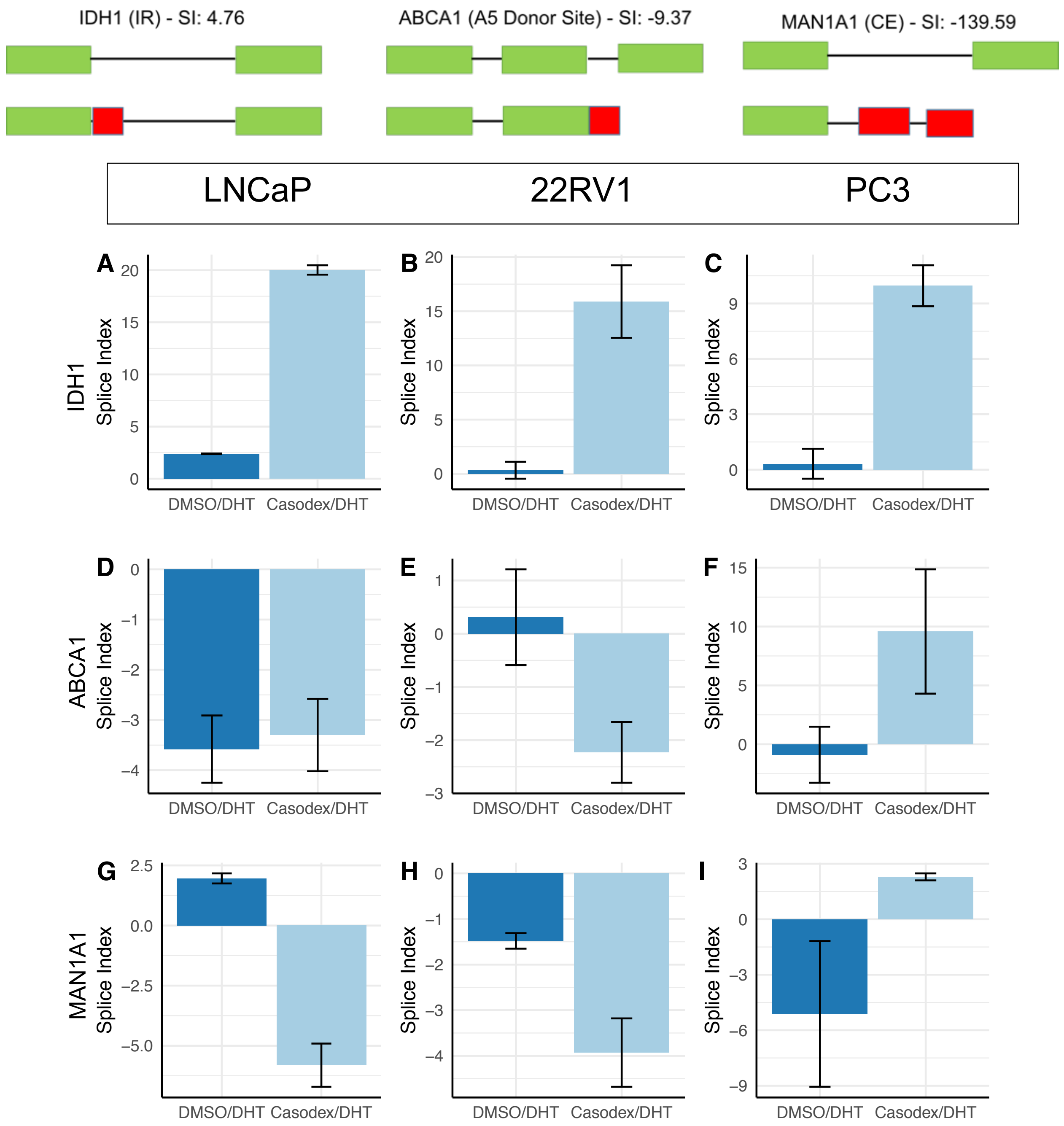

Supplementary Figure 4

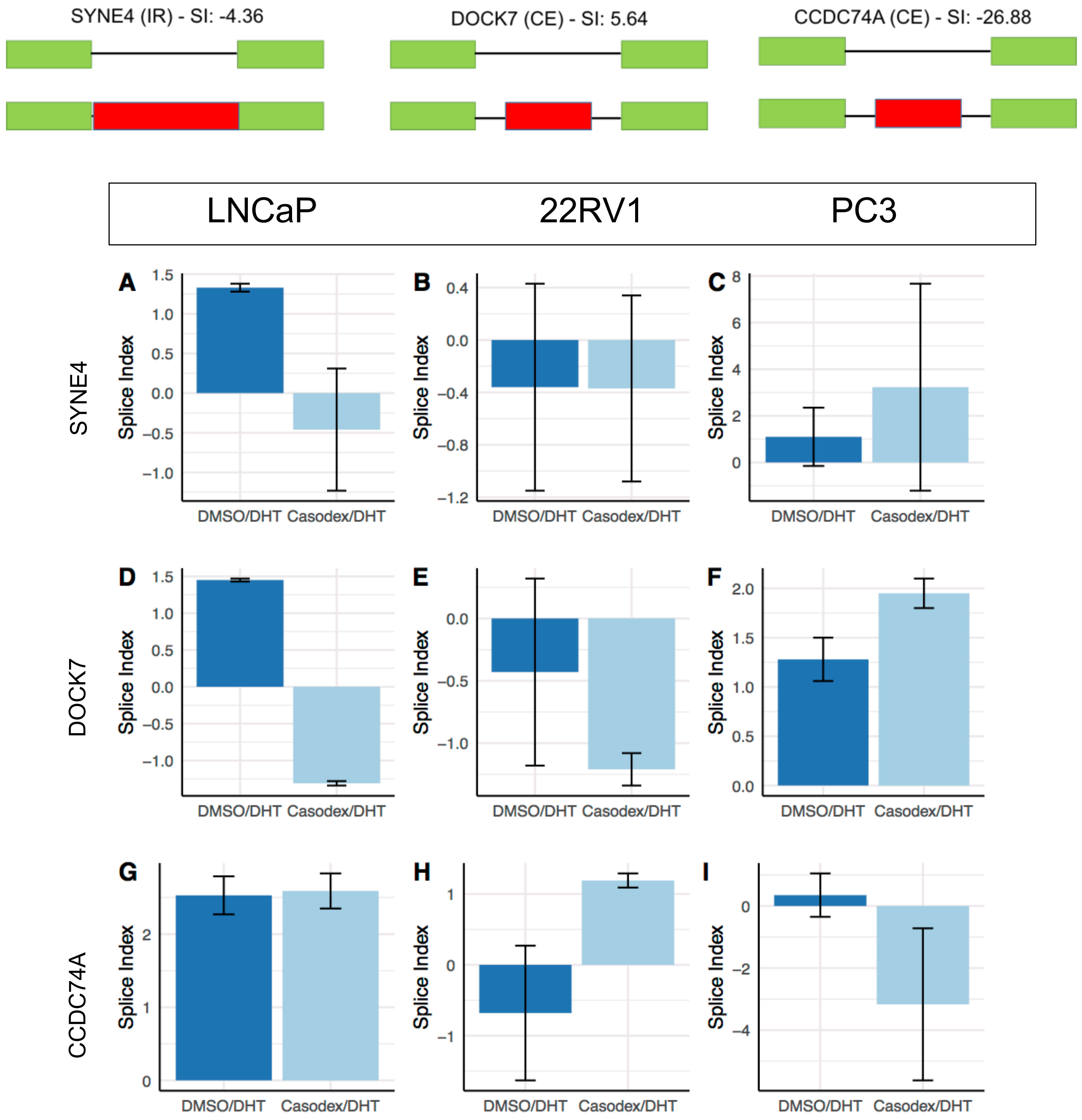

Supplementary Figure 5

0 **A**

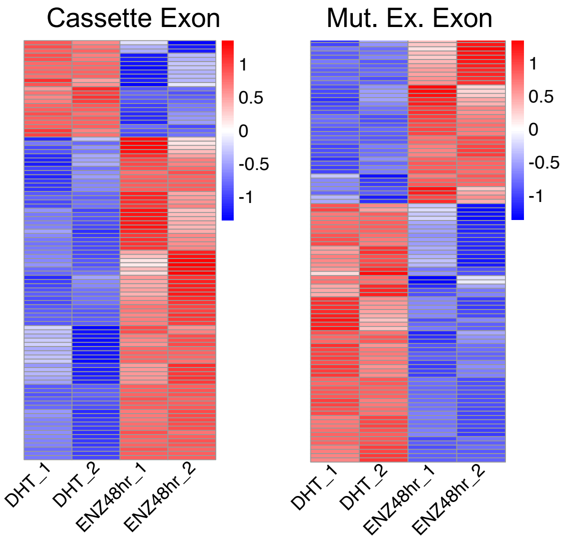

B

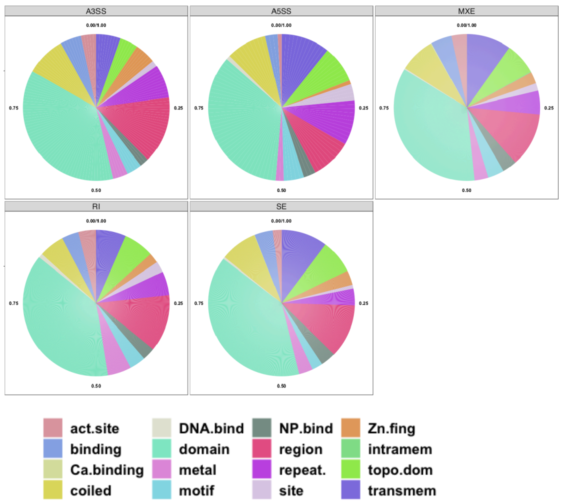

C

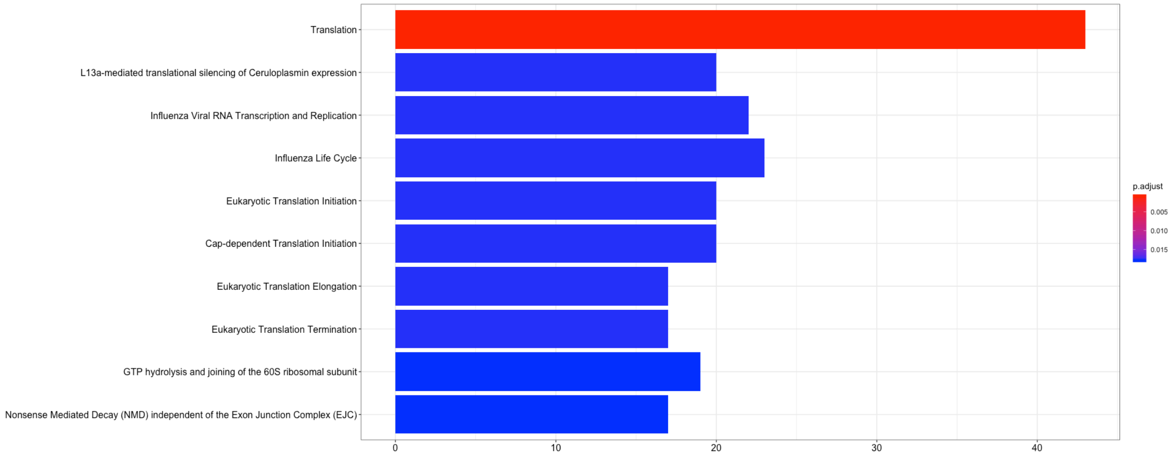

Supplementary Figure 5

D

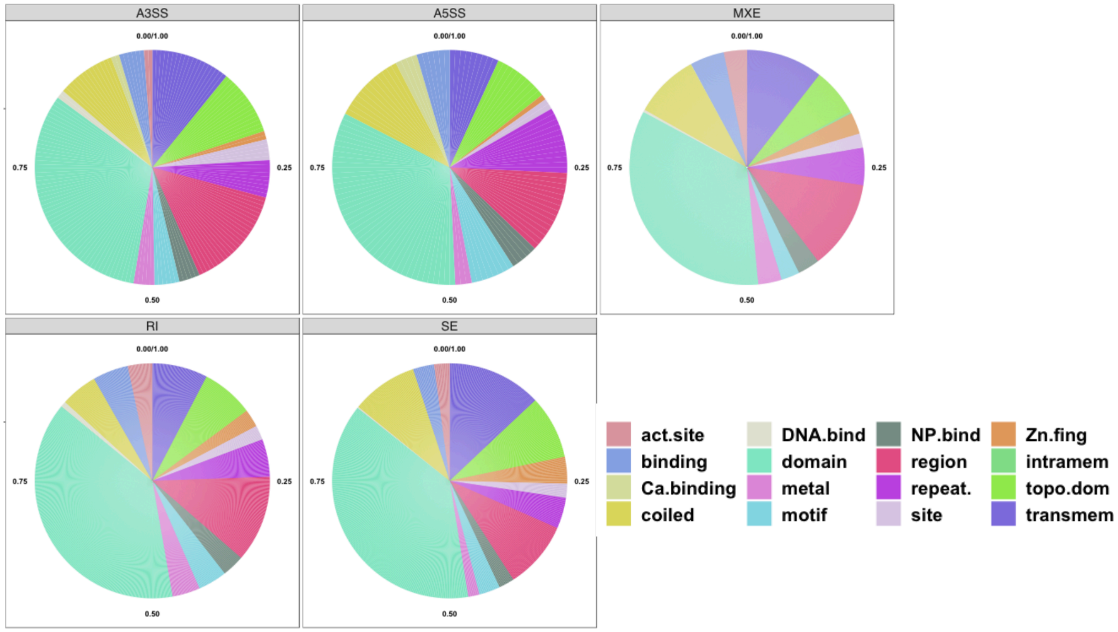

E

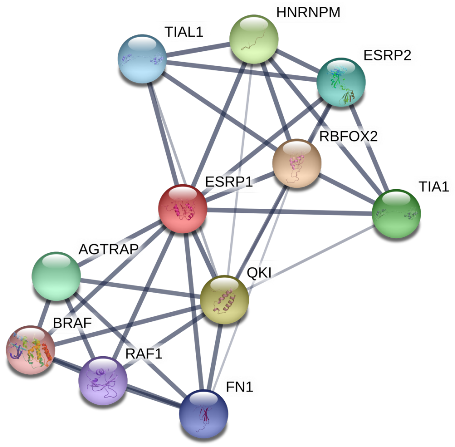

	10uM Casodex Vs 10nMDHT		
Gene Symbol	Fold Change	P-val	FDR P-val
ESRP2	-4.91	9.78E-10	5.46E-07
ESRP1	-1.56	2.72E-06	7.00E-05
RBFOX2	1.46	3.11E-05	0.0004
TIA1	1.3	0.0009	0.0057
RAF1	-1.28	0.0009	0.0058
AGTRAP	-1.32	0.001	0.0065
BRAF	1.46	0.0025	0.0131
FN1	-1.15	0.0332	0.1018
TIAL1	1.04	0.6653	0.7961
HNRNPM	-1.04	0.7528	0.8561

Supplementary Figure 5

F

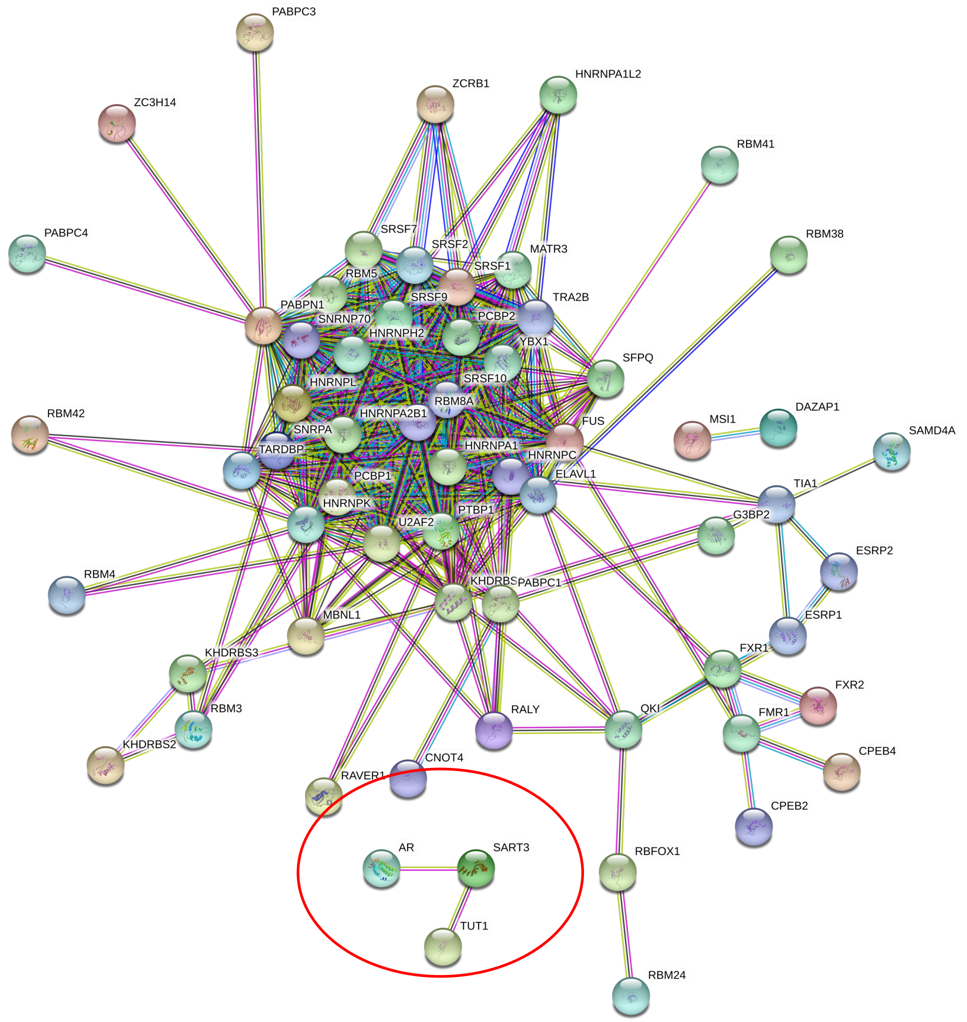

G

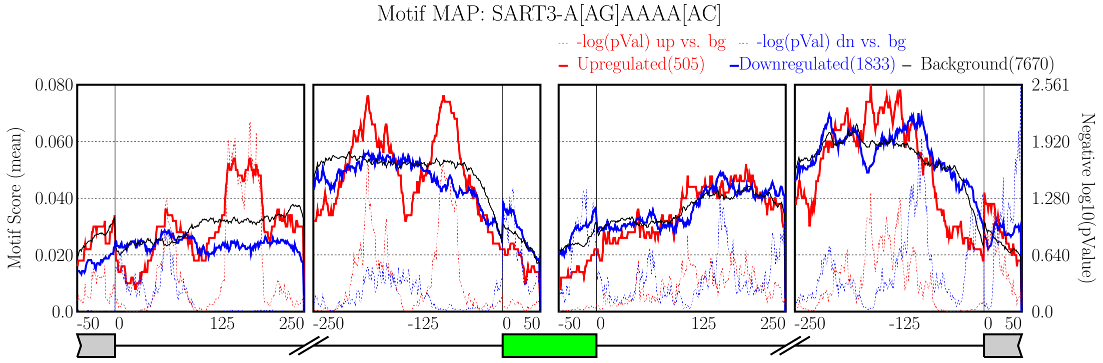

Supplementary Figure 6

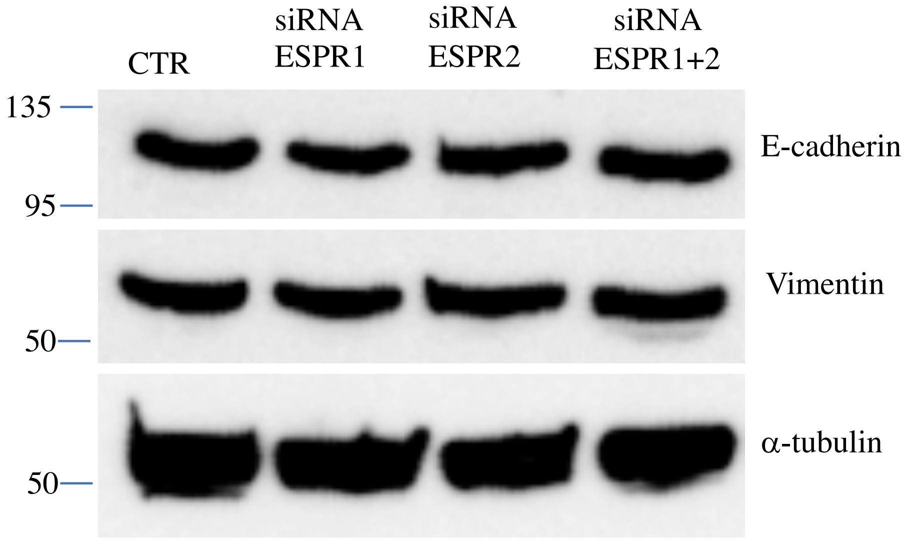

PC3

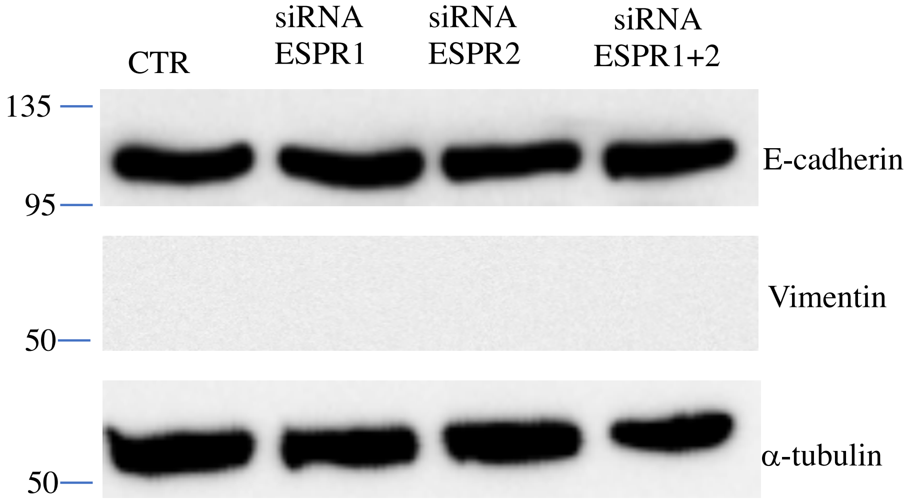

22RV1

Supplementary Figure 7

A

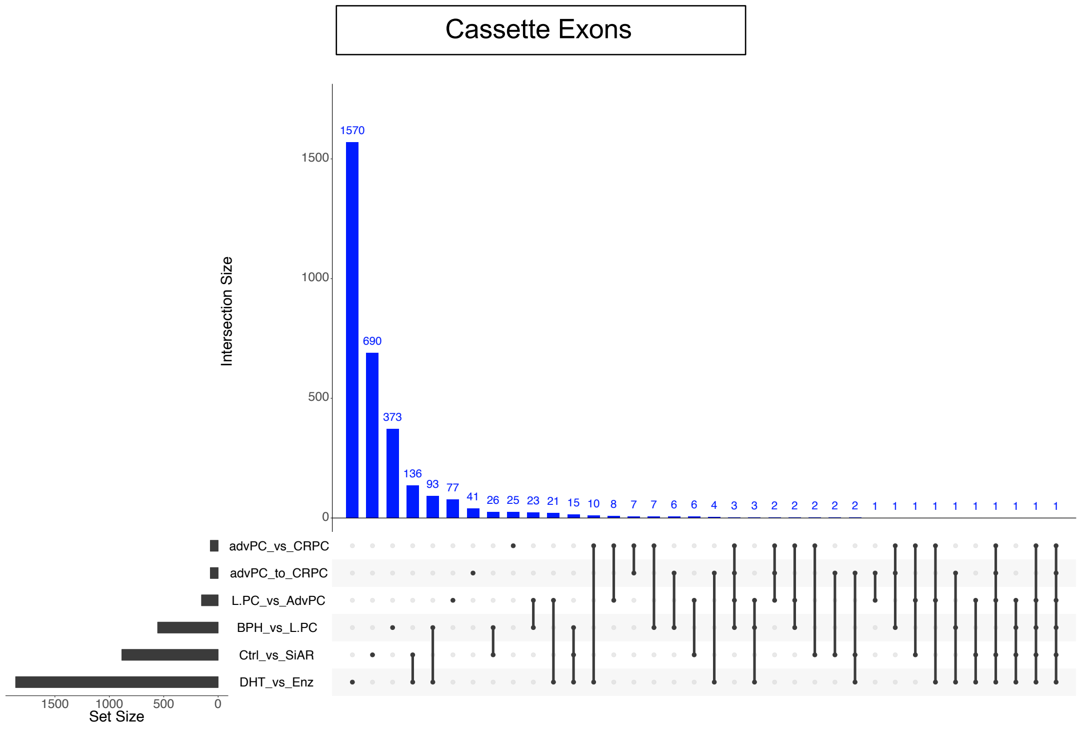

B

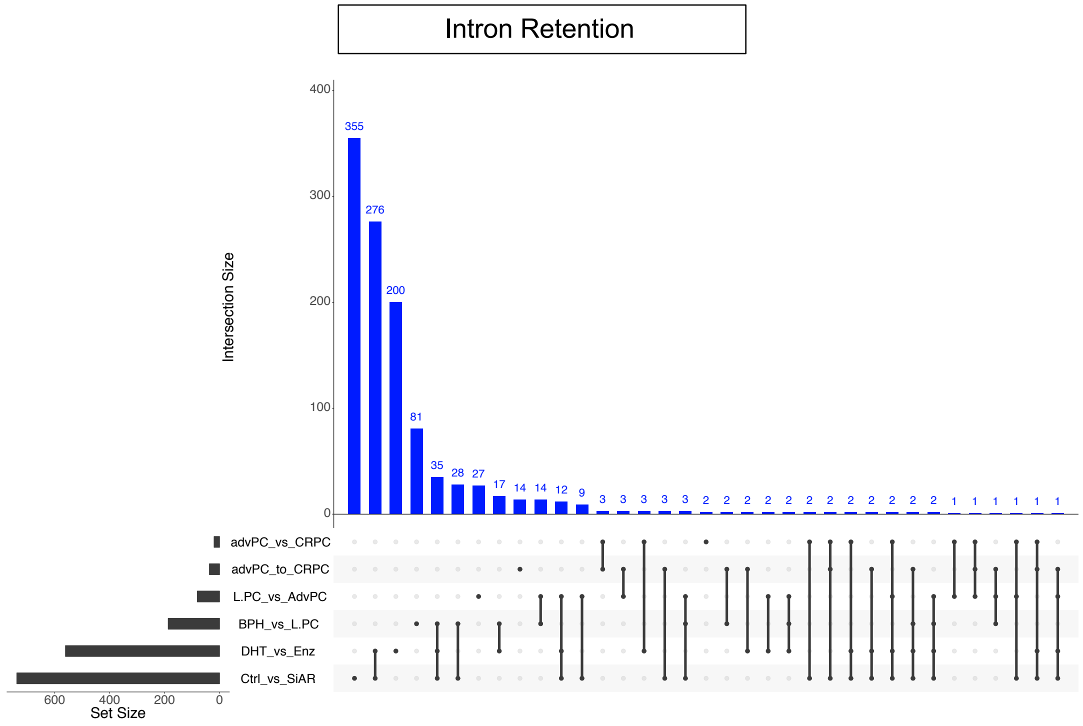

Supplementary Figure 7

C

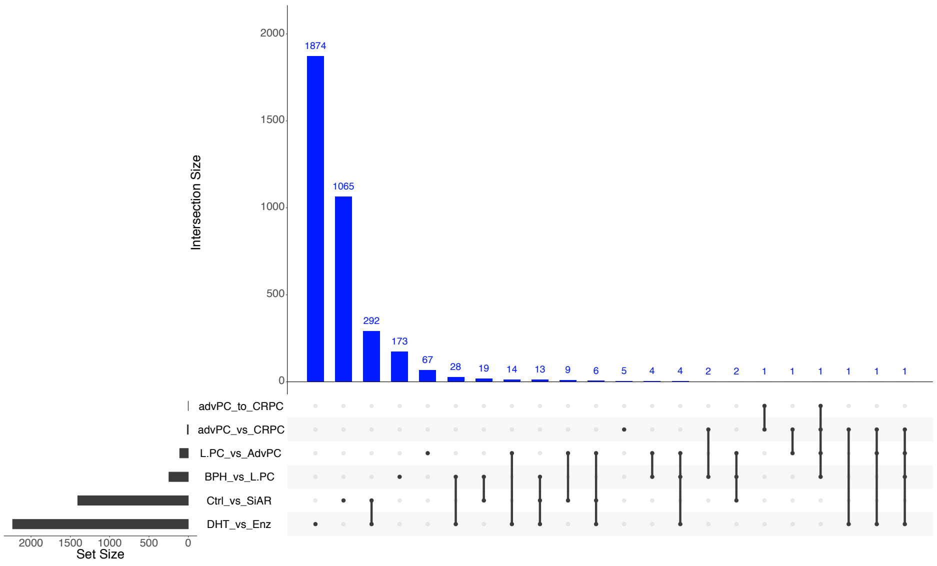

D

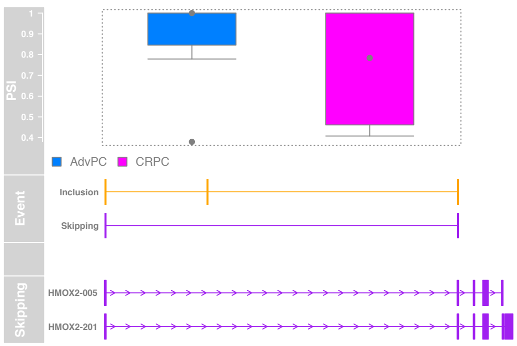

E

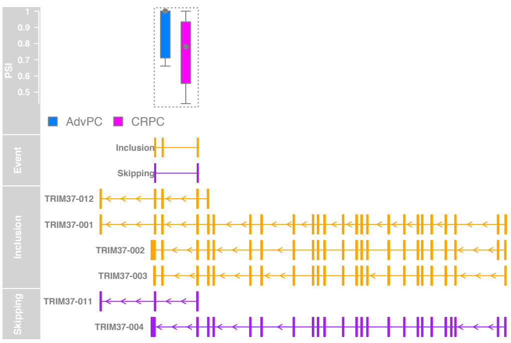

F

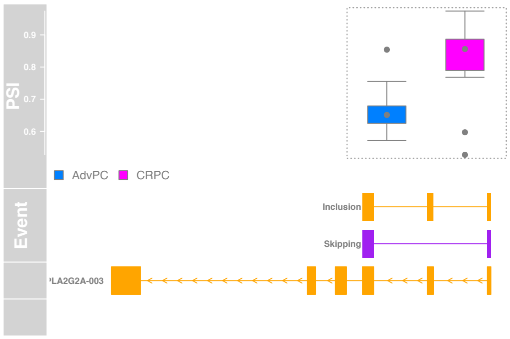

G

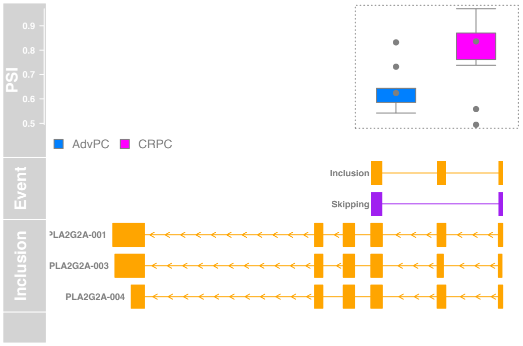

Supplementary Figure 7

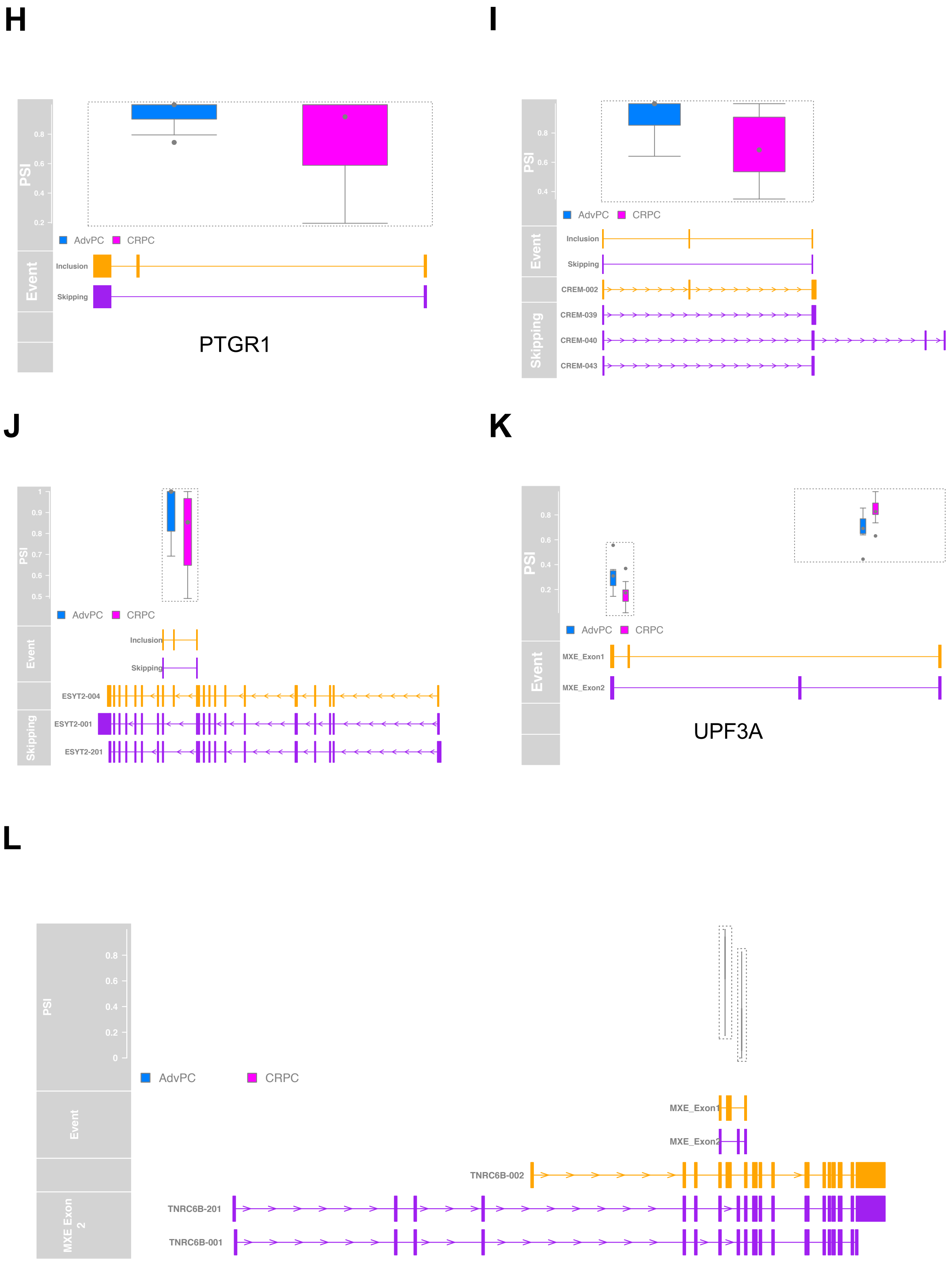

Supplementary Figure 7

M

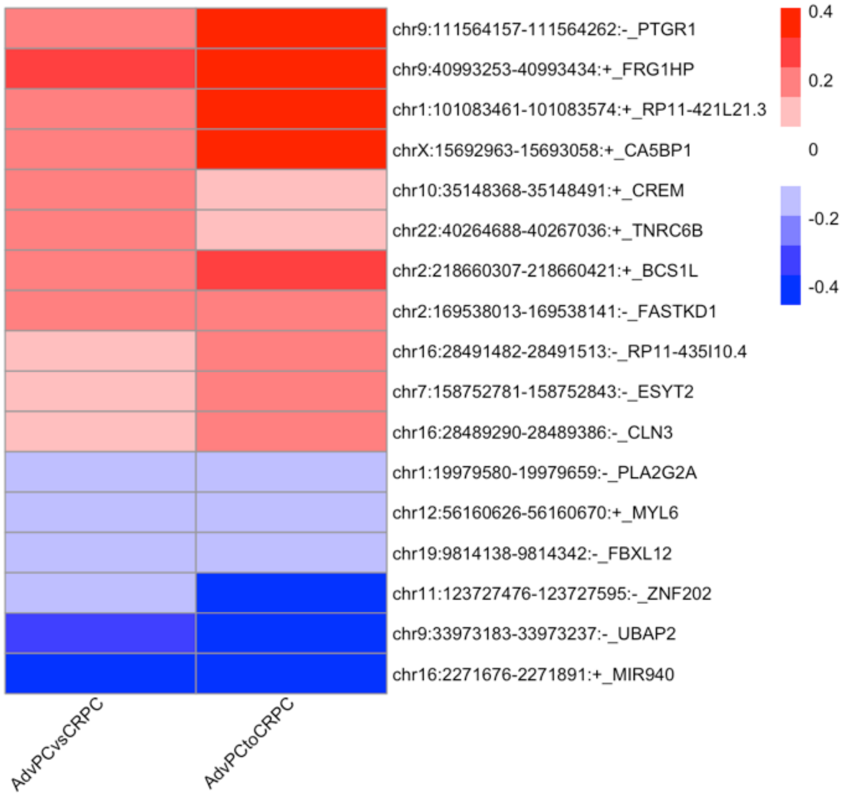

Supplementary Figure and Table Legend

Supplementary Figure 1: Analytical schematic to identify androgen regulated alternative splicing that may be associated with prostate cancer progression

Supplementary Figure 2–4: RT-PCR validation of alternative splicing events predicted using splicing array. Bar-graph showing the average splice index of genes including RANBP3L, AAK1, TTBK2, IDH1, ABCA1, MAN1A1, SYNE4, DOCK7, and CCDC74A across LNCaP, 22RV1, and PC3 cells from RT-PCR data calculated using the $\Delta\Delta$Ct method relative to an endogenous reference gene (HPRT or GAPDH).

Supplementary Figure 5: (A) The heatmap comparing showing top–100 significantly different PSI for MEE and CE across different samples including LNCaP cells treated with DHT or enzalutamide. (B) Pie-charts show the frequency of splicing events induced by enzalutamide in comparison to DHT treatment of LNCaP cells that are mapping to the protein functional domains including transmembrane domain, coiled region, topo domain, metal binding, zinc finger binding, activation site for protein, and others. (C) Bar-graph showing the GO pathways enriched for a common set of genes undergoing alternatively splicing in response to treatment with DHT, enzalutamide or siRNA against AR. (D) Pie-charts show the frequency of splicing events induced by the genomic inhibition of AR in MDA-PCa-2B cells that are mapping to the functional protein domain. (E) Protein-protein network analysis shows ESRP1 and ESRP2 binding proteins. The thickness of edges

represents confidence in protein-protein associations. The table shows the differential expression (10µM casodex vs 10nm DHT) for ESRP1 and ESRP2 binding partners.

Supplementary Figure 6: PC3 and 22 RVI cells were transfected with indicated siRNA, after 24 h, western blotting of EMT markers (E-cadherin, and Vimentin) was performed using Tubulin as loading control.

Supplementary Figure 7: (A-C) Upset plot showing an overlap and unique cassette exon, Intron retention, and mutually exclusive events across comparison groups including patients with BPH, L.PC, AdvPC, CRPC, LNCaP cells treated including DHT or enzalutamide, MDA-PCa-2b cells treated with scrambled siRNA or siRNA. (D-L) A combined transcript plot including a box plot comparing the PSI between patients with AdvPC and CRPC, the schematic of the splicing event, and predicted ensembl transcript plot for cancer-relevant genes. (M) Heatmap comparing the PSI for the ASE predicted to be common across AdvPC vs. CRPC and a longitudinal comparison within patients who progressed from AdvPC to CRPC.

Supplementary Table 1: Table of differentially expressed genes in LNCaP cells that were cultured for 72 hours in charcoal-stripped fetal bovine serum and stimulated with DMSO, 10nM DHT, or 10µM casodex for 24 hours.

Supplementary Table 2: Table of differentially spliced genes in LNCaP cells that were cultured for 72 hours in charcoal-stripped fetal bovine serum and stimulated with DMSO, 10nM DHT, or 10µM casodex for 24 hours.

Supplementary Table 3: List of primers and their sequences used for validating splicing events predicted by HTA-2.0 analysis.

Supplementary Table 4: Raw data for RT-PCR assay validating HTA-2.0 predicted splicing events in LCNaP, 22RV1, and PC3 cells respectively. Each sheet represents three sets of biological replicates performed with three set of technical replicates.

Supplementary Table 5: List of curated 100 genes that are associated with prostate cancer progression and development.
